# Síndrome de fuga capilar sistémica: Un caso con presentación severa y respuesta al tratamiento

**DOI:** 10.23938/ASSN.1141

**Published:** 2025-10-16

**Authors:** Ignacio Lasierra Lavilla, Cherpentier Fonseca López, Isabel Ester Escuer Núñez, Francisco Javier Lerín Sánchez

**Affiliations:** 1 Servicio de Medicina Interna Hospital Obispo Polanco Servicio Aragonés de Salud Teruel España; 2 Servicio de Endocrinología Hospital Obispo Polanco Servicio Aragonés de Salud Teruel España

**Keywords:** Síndrome de Fuga Capilar, Hemoconcentración, Hipoalbuminemia, Síndrome compartimental, Inmunoglobulinas, Capillary Leak Syndrome, Hemoconcentration, Hypoalbuminemia, Compartment Syndrome, Immunoglobulins

## Abstract

El síndrome de fuga capilar sistémica es una enfermedad rara, descrita por primera vez en 1960, caracterizada por episodios recurrentes de shock, hemoconcentración e hipoalbuminemia. Su diagnóstico es clínico y su manejo incluye medidas de soporte y tratamiento con inmunoglobulinas. Se trata de un cuadro potencialmente mortal, por lo que su diagnóstico precoz y un adecuado manejo resulta fundamental para mejorar el pronóstico.

Se presenta el caso de un varón de 48 años con episodios de shock, hemoconcentración e hipoalbuminemia, inicialmente sugestivo de sepsis. Requirió tratamiento intensivo y fasciotomías de las cuatro extremidades por síndrome compartimental, siendo diagnosticado de síndrome de fuga capilar sistémica tras un segundo ingreso. Se instauró tratamiento con inmunoglobulinas, teofilina y salbutamol con buena respuesta hasta la actualidad.

## Abstract (en)

Systemic capillary leak syndrome is a rare disorder, first described in 1960, characterized by recurrent episodes of shock, hemoconcentration, and hypoalbuminemia. Diagnosis is primarily clinical, and management includes supportive care and immunoglobulin therapy. Given its potentially life-threatening course, early recognition and timely treatment are essential to improving outcomes.

We report the case of a 48-year-old man with recurrent episodes of shock, hemoconcentration, and hypoalbuminemia, initially misdiagnosed as sepsis. He required intensive care management and underwent fasciotomies of all four extremities due to compartment syndrome. During the second hospitalization, he was correctly diagnosed with systemic capillary leak syndrome. Treatment with intravenous immunoglobulins, theophylline, and salbutamol was initiated, leading to a sustained favorable clinical response to date.

## INTRODUCCIÓN

El síndrome de fuga capilar sistémica, también conocido como enfermedad de Clarkson, es una patología rara y potencialmente mortal descrita por primera vez en 1960 por Clarkson y col^1^. Se caracteriza por episodios recurrentes de shock, hemoconcentración e hipoalbuminemia, secundarios a la fuga de plasma y proteínas al espacio extravascular. Hasta la fecha, se han reportado aproximadamente 500 casos, lo que refleja su baja prevalencia y la dificultad de su diagnóstico^2^^,^^3^. Su reconocimiento es fundamental debido a su elevada mortalidad y a la posibilidad de pasar desapercibido en los estadios iniciales.

El diagnóstico es clínico y su tratamiento agudo consiste en medidas de soporte^4^. Las inmunoglobulinas pueden reducir la frecuencia de los brotes y mejorar la supervivencia^2^^,^^3^.

Presentamos el caso de un varón de 48 años quien, tras un segundo ingreso por shock, hemoconcentración e hipoalbuminemia, fue diagnosticado de síndrome de fuga capilar sistémica, debutando de una forma atípica y con un brote inicial de gran severidad, que precisó fasciotomía de las cuatro extremidades por síndrome compartimental.

## CASO CLÍNICO

Presentamos el caso de un varón de 48 años, sin antecedentes médicos de interés, que en marzo de 2023 acudió a urgencias por dolor torácico irradiado a espalda y episodio presincopal. Los días previos había presentado clínica catarral. Durante la exploración, destacó una presión arterial de 70/40 mm Hg y signos de hipoperfusión distal. Se administró fluidoterapia intensa, consiguiendo aumentarla a 110/72 mm Hg. Inicialmente, se sospechó disección aórtica, que se descartó mediante angio-TC (tomografía cumputarizada). Tras nuevo descenso de la presión arterial, se decidió su ingreso en la unidad de Cuidados Intensivos (UCI).

En UCI se inició tratamiento con fluidos y noradrenalina. A las pocas horas tras el ingreso presentó inflamación y dolor intenso en las cuatro extremidades, compatible con síndrome compartimental, que requirió fasciotomías de las cuatro extremidades. Inicialmente, ante la sospecha de shock séptico, se inició tratamiento empírico con meropenem y daptomicina, aunque el paciente no presentaba fiebre ni elevación de reactantes de fase aguda, y los estudios microbiológicos eran negativos. Posteriormente, ante la posibilidad de un shock tóxico, se inició tratamiento con inmunoglobulinas, lo que permitió la retirada progresiva de la noradrenalina y el alta hospitalaria. Las analíticas realizadas durante el ingreso mostraban un deterioro progresivo de la función renal, acidosis metabólica, cifras elevadas de hemoglobina y hematocrito, e hipoproteinemia con hipoalbuminemia, que se normalizaron posteriormente (Tabla 1).

En noviembre de 2023, el paciente acudió nuevamente a urgencias por malestar general y mareo. Presentó disminución de la presión arterial y edema en extremidades inferiores. La analítica realizada en urgencias (Tabla 2) mostró hemoglobina elevada (23,2 g/dL) y deterioro de la función renal. Se instauró tratamiento de soporte y diurético, con una excelente respuesta a las 72 horas. Además, durante su ingreso se realizaron pruebas que descartaron un posible síndrome mieloproliferativo (ausencia de mutación JAK2 V617F), causas infecciosas (hemocultivos y sedimento urinario negativo) y un estudio analítico descartando enfermedad autoinmune. También se solicitaron valores de complemento C1q, y de cortisol, descartando un posible angioedema y una insuficiencia suprarrenal. El proteinograma evidenció la presencia de una gammapatía monoclonal de significado incierto (GMSI).

**Tabla 1:** Evolución analítica durante el primer ingreso

	Valores analíticos				
Parámetro	Al ingreso	24 h	48 h	72 h	Al alta
Creatinina (mg/dL)	1,53	1,58	3,15	1,42	0,7
Proteínas (g/dL)	6,7	2,7	2,5	4	6
Albúmina (g/dL)	2,3	1,2	1,1	1,8	2,9
Leucocitos (10^9^/L)	24,54	51,09	21,16	9,21	8,23
Hemoglobina (g/dL)	25,8	20,8	0	8	12,1
Hematocrito (%)	71,4	58,4	8	22,2	34,4
Plaquetas (10^9^/L)	388	288	131	58	146

**Tabla 2:** Evolución analítica durante el segundo ingreso

	Valores analíticos				
Parámetro	Al ingreso	24 h	48 h	72 h	Al alta
Creatinina (mg/dL)	1,48	1,65	0,98	0,76	0,81
Proteínas (g/dL)	4,2	4,5	4,9	5,4	6,8
Albúmina (g/dL)	2,3	2,1	2,8	2,9	
Leucocitos (10^9^/L)	20,04	32,33	8,41	4,21	4,36
Hemoglobina (g/dL)	23,2	22,2	13	11	13,4
Hematocrito (%)	61,6	59,4	35,7	30,6	37,1
Plaquetas (10^9^/L)	343	393	193	132	192

Con estos hallazgos se estableció el diagnóstico de síndrome de fuga capilar sistémica idiopático, iniciando tratamiento con inmunoglobulinas, teofilina y salbutamol. El paciente ha permanecido sin nuevos brotes desde el inicio del tratamiento.

## DISCUSIÓN

El síndrome de fuga capilar sistémica es una enfermedad poco frecuente y potencialmente grave. La frecuencia e intensidad de los brotes varía, lo que retrasa y dificulta su diagnóstico^4^. Suele afectar a adultos de mediana edad y no existe predominio por sexo^3^. El diagnóstico es de exclusión, sin tener una definición o criterios establecidos, caracterizándose en la práctica clínica por la tríada de disminución de la presión arterial - habitualmente acompañada de edema -, hemoconcentración e hipoalbuminemia^4^. Nuestro paciente presentó estos síntomas en ambos ingresos.

La fisiopatología no está establecida, algunos estudios han detectado durante los brotes elevación de mediadores proinflamatorios como la interleuquina-6 (IL-6) o la proteína quimioatrayente de monocitos 1 (CCL2), factores de permeabilidad angiogénica como el factor de crecimiento endotelial vascular (VEGF) o la angiopoyetina 2 (ANG-2), así como elevación del óxido nítrico (NO), con normalización posterior de sus niveles tras la resolución del brote^3^. Se ha descrito una asociación entre el síndrome de fuga capilar sistémica idiopático y la presencia de GMSI^3^^,^^4^^,^^5^, como en nuestro paciente, relacionándose un alto componente monoclonal al diagnóstico con un mayor riesgo de nuevos brotes, aunque sin impacto en el pronóstico^5^.

La evolución clínica consta tres fases: prodrómica, de fuga y de reabsorción.

- La fase prodrómica se caracteriza por malestar general, astenia y debilidad^4^. Los factores que actúan como desencadenantes de esta fase más frecuentemente (hasta en el 40-50% de los casos)^2^^,^^3^^,^^6^^-^^8^ son las infecciones, mayoritariamente víricas (gripe, virus sincitial respiratorio, *Influenza* o SARS-CoV-2). Otros posibles desencadenantes, aunque menos frecuentes, pueden ser un esfuerzo intenso, el estrés o algunos fármacos^3^^,^^9^ (Tabla 3). Nuestro paciente presentó un cuadro catarral los días previos al ingreso y había recibido recientemente la vacuna para el SARS-CoV-2, hecho también descrito en los últimos años como posible desencadenante^10^.
- La fase de fuga se caracteriza por el rápido desarrollo de shock con edematización secundaria a la fuga plasmática y de proteínas hacia el espacio extravascular; suele durar entre uno y cuatro días. Pueden surgir varias complicaciones (Tabla 3), como insuficiencia renal aguda de origen prerrenal^4^, síndrome compartimental^9^, rabdomiólisis, trombosis secundarias a la hemoconcentración^3^^,^^11^^)^ o fracaso multiorgánico^3^, por lo que es la fase con una mayor mortalidad^2^. El paciente presentado sufrió insuficiencia renal aguda en ambos ingresos, y durante el primero precisó una intervención quirúrgica urgente de las cuatro extremidades por un síndrome compartimental, siendo muy poco frecuente este tipo de presentación tan grave.
- La fase de reabsorción ocurre por la vuelta del líquido al espacio intravascular, con normalización de la presión arterial y una abundante diuresis. Debido a la fluidoterapia administrada previamente, durante esta fase existe el riesgo de desarrollar edema agudo pulmonar^3^ (Tabla 3).

**Tabla 3:** Desencadenantes y complicaciones presentadas en otros casos clínicos y características de pacientes

Autor (Año)	Edad (años)	Sexo	Desencadenante	Complicaciones
Miyazawa y col^4^ 2024	60	H	-	IRA prerrenal
Naito y col^6^ 2023	62	H	Infección (SARS CoV-2)	IRA prerrenal
				PCR
				EAP
Zec y col^7^ 2022	68	H	Infección (*Influenza* B)	DP
				EAP
				Rabdomiólisis
Kasugai y col^9^ 2020	18	M	Sobredosis (metformina e iDPP4)	Rabdomiólisis
Síndrome compartimental			
Correia y col^11^ 2019	48	H	-	IRA prerrenal
Isquemia mesentérica			
Raith y col^12^ 2018	27	H	-	IRA prerrenal
Síndrome compartimental			

H: hombre; M: mujer; -: desconocido; iDPP4: inhibidor de la dipeptidil peptidasa IV; IRA: insuficiencia renal aguda; PCR: parada cardiorrespiratoria; EAP: edema agudo de pulmón; DP: derrame pericárdico; Tto: tratamiento; Ig: inmunoglobulinas.

Se realizó una búsqueda bibliográfica en PubMed (“*systemic capillary leak syndrome*” *AND* “*complications*”) y se obtuvieron 59 resultados en los últimos diez años. La Tabla 3 presenta los seis casos que presentaron las complicaciones más llamativas.

Es importante descartar otras entidades que pueden tener una presentación similar, como un shock séptico mediante estudios microbiológicos, una *policitemia vera* mediante un análisis genético, un angioedema hereditario solicitando niveles de complemento C1q, o un síndrome nefrótico realizando un sedimento urinario^3^^,^^12^. Estas pruebas resultaron negativas en el paciente presentado.

El tratamiento durante los brotes consiste en medidas de soporte, con un adecuado manejo de fluidoterapia, fármacos vasoactivos y profilaxis de trombosis. No se ha demostrado beneficio del tratamiento durante la fase aguda con corticoides, anticuerpos monoclonales u otros antiinflamatorios^3^. Sin embargo, varios estudios han observado el beneficio del tratamiento con inmunoglobulinas, tanto durante los brotes agudos como especialmente en su prevención, consiguiendo una importante disminución en frecuencia e intensidad de los brotes y en mortalidad de los pacientes tratados mensualmente con inmunoglobulinas^2^^,^^5^^,^^13^. Solo uno de los casos mostrados en la Tabla 3 no recibió tratamiento con inmunoglobulinas^9^; los otros cinco casos mostraron respuesta a dicho tratamiento. La teofilina y los beta-2-adrenérgicos puede ayudar durante la fase aguda y en la prevención de nuevos episodios^3^^,^^14^. Tras el segundo ingreso, a nuestro paciente se le pautó tratamiento con inmunoglobulinas mensuales, teofilina y salbutamol, sin volver a presentar nuevos brotes desde el inicio del tratamiento.

En conclusión, el síndrome de fuga capilar sistémica es una enfermedad rara y potencialmente mortal caracterizada por episodios de shock, hemoconcentración e hipoalbuminemia. Probablemente infradiagnosticada, su reconocimiento temprano es clave para un manejo adecuado y la prevención de complicaciones. Este caso resalta la importancia del diagnóstico diferencial preciso, la gravedad de sus posibles complicaciones y el rol esencial de las inmunoglobulinas en la reducción de brotes y la mejora del pronóstico.

## Data Availability

Se encuentran disponibles bajo petición al autor de correspondencia.
